# Ultra-high-resolution mapping of ambient fine particulate matter to estimate human exposure in Beijing

**DOI:** 10.1038/s43247-023-01119-3

**Published:** 2023-12-01

**Authors:** Yongyue Wang, Qiwei Li, Zhenyu Luo, Junchao Zhao, Zhaofeng Lv, Qiuju Deng, Jing Liu, Majid Ezzati, Jill Baumgartner, Huan Liu, Kebin He

**Affiliations:** 1 State Environmental Protection Key Laboratory of Sources and Control of Air Pollution Complex, State Key Joint Laboratory of Environmental Simulation and Pollution Control, School of Environment, Tsinghua University, Beijing 100084, China; 2 Centre for Clinical and Epidemiologic Research, Beijing An Zhen Hospital, Capital Medical University, Beijing Institute of Heart, Lung and Blood Vessel Diseases, Beijing 100029, China; 3 School of Public Health, Imperial College London, London SW72AZ, UK; 4 School of Population and Global Health, McGill University, Montréal, QC H3A0G4, Canada

## Abstract

With the decreasing regional-transported levels, the health risk assessment derived from fine particulate matter (PM_2.5_) has become insufficient to reflect the contribution of local source heterogeneity to the exposure differences. Here, we combined the both ultra-high-resolution PM_2.5_ concentration with population distribution to provide the personal daily PM_2.5_ internal dose considering the indoor/outdoor exposure difference. A 30-m PM_2.5_ assimilating method was developed fusing multiple auxiliary predictors, achieving higher accuracy (R^2^ = 0.78–0.82) than the chemical transport model outputs without any post-simulation data-oriented enhancement (R^2^ = 0.31–0.64). Weekly difference was identified from hourly mobile signaling data in 30-m resolution population distribution. The population-weighted ambient PM_2.5_ concentrations range among districts but fail to reflect exposure differences. Derived from the indoor/outdoor ratio, the average indoor PM_2.5_ concentration was 26.5 μg/m^3^. The internal dose based on the assimilated indoor/outdoor PM_2.5_ concentration shows high exposure diversity among sub-groups, and the attributed mortality increased by 24.0% than the coarser unassimilated model.

Anthropogenic air pollution stemming from rapid economic development and industrialization is a significant global public health issue^1–3^, causing approximately 7 million premature deaths worldwide each year^4^. Fine particulate matter (particles that are 2.5 microns or less in aerodynamic equivalent diameter, PM_2.5_), as a major air pollutant, has been proved to be linked to many diseases^5–7^. Several exposure assessment models were developed to estimate the premature mortality attributed to PM_2.5_ exposure, linking the exposure effects directly to the ambient pollution level^8,9^. The ambient PM_2.5_ levels was once sufficed to assess the significant difference in PM_2.5_ exposure between cities in the earlier years^10–14^, and the contribution of spatial heterogeneity from local sources to the exposure differences was negligible at such high ambient levels^15–18^. However, with the ambient PM_2.5_ level declining sharply^19–22^, the contribution of local PM_2_._5_ sources has grown to 50–80%^23–25^. The contribution of spatial heterogeneity from local sources to population exposure has become increasingly pronounced. PM_2.5_ levels in some micro-environments can still be high^26–28^, up to hundreds of micrograms per cubic meter^29,30^. Solely adopting ambient PM_2.5_ levels is no longer enough to evaluate the exposure differences caused by local sources.

A more accurate way to assess PM_2.5_ exposure is by measuring the personal internal dose. It reflects the cumulative interaction between human physiological activities and environmental pollution^31–33^. Direct approaches involving portable monitoring suits can effectively obtain the personal real-time exposure^34,35^, but the cost highly limits its application in extensive sampling. Indirect approaches, estimated from the observed or simulated PM_2.5_ distribution, are more suitable for obtaining adequate samples at the city level^36–38^. However, the monitoring value is only representative in the adjacent area^39^, and the chemical transport models (CTMs) can usually reach a resolution of at most 1 km^40–42^, making it all challenging to identify exposure differences among micro-environments. The development of ultra-high-resolution concentration field can effectively improve the population coverage and granularity together with the high-resolution population activity data.

To derive an ultra-high-resolution PM_2.5_ distribution with better accuracy, auxiliary data sets have been fused with the observation data in recent studies, such as satellite measurements, CTM outputs, and meteorological variables^43,44^. However, the model resolution can sometimes still be limited, and some ultra-high-resolution variables (30-m land use or elevation) had to be resampled to a coarser level (i.e., 1 km) for match to avoid additional uncertainties^45,46^. Until recently, the availability of a vast amount of ultra-high-resolution data^47^ like aerosol optical depth (AOD) data from Sentinel-2A satellite and top of atmosphere (TOA) data from Landsat-8 satellite^48,49^ has made it no longer an obstacle for air quality applications. The linear land use regression (LUR) model has been used to fuse these auxiliary variables^50–53^. However, as the relationship between PM_2.5_ and auxiliary variables is usually nonlinear and complex^54^, the machine learning methods were introduced, achieving better performance and considerable extrapolation robustness^55–57^. The incorporation of both multiple ultra-high resolution data and machine learning algorithms is more likely to gain better performance than taking only a single one of them.

Here, the overall research process of this study is briefly shown in Fig. 1, including performing the CTM model, ultra-high resolution assimilation, population exposure, personal indoor exposure, and health risk assessment. By fusing ultra-high-resolution multisource auxiliary data, we simulated the street-level distribution of PM_2.5_, and combine it with the ultra-high-resolution population activity data to calculate the personal internal dose based on considering the indoor/outdoor (I/O) exposure differences. The mortality burden assessment was performed to show the difference between the city-level ambient PM_2.5_ distribution derived from 30-m and 1-km resolution result without any further consideration of the I/O exposure patterns, for the existing exposure-response relationship was only based on the ambient PM_2.5_ level. The potential of this method for exposure inequality identification and health risk assessment was discussed.

## Results and discussions

### Ultra-high resolution PM_2.5_ mapping in Beijing

**Fig. 2:**
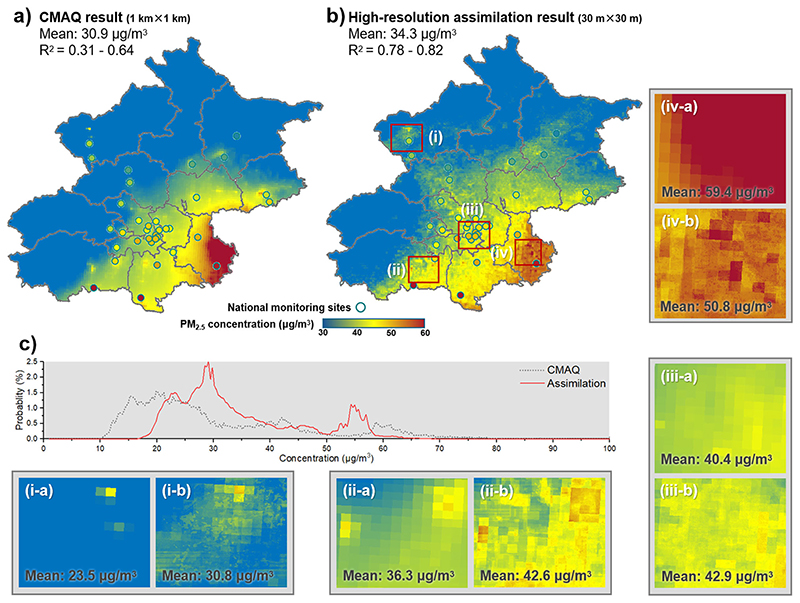
Comparison of simulated PM_2.5_ concentration before and after assimilation. **a**, **b** Model performance and mapping comparison among Beijing from WRF-CMAQ model, our method, and monitoring data, with 4 extracted local areas (i) – (iv) for detailed difference. **c** Probability distribution comparison of PM_2.5_ concentration intervals.

Assimilated by other auxiliary variables, the ultra-high resolution PM_2.5_ concentration distribution was mapped throughout Beijing based on the WRF-CMAQ simulation output (annual average in Fig. 2 and seasonal result in Supplementary Fig. 3). With the increase of the resolution and the monitoring-data-based assimilation, the 30-m mapping achieved higher accuracy (R^2^ = 0.78–0.82) to the national monitoring data, compared with the WRF-CMAQ output (R^2^ = 0.31–0.64), resulting in the annual average increased from 30.9 μg/m^3^ to 34.3 μg/m^3^. In the PM_2.5_ concentration probability density distribution curve, the peaks at 10–30 μg/m^3^ and 55–70 μg/m^3^ intervals moved to the 20–40 μg/m^3^ and 55–70 μg/m^3^, respectively. It indicated that the overestimation in the southeastern and underestimation in the northwestern were both greatly amended. Area (i) showed a 30.84% increase, and area (iv) showed a 16.92% decrease. The areas (ii) and (iii) with moderate concentration variations but with local hotspots effectively identified could not be achieved by the WRF-CMAQ model on its own. Furthermore, the original concentration probability density distribution curve from WRF-CMAQ was quite zigzag, while became smoother after improving the spatial resolution, for the changes between the adjacent coarse grids are more finely transited by the smaller grids. It also showed that while maintaining the overall spatial distribution trend, the description of far more detailed information on a finer scale was effectively achieved after conducting the comparison result of two-dimensional discrete Fourier transform and high pass filtering on the PM_2.5_ concentration distribution picture (Supplementary Method; Supplementary Discussion 2; Supplementary Fig. 4).

As the output of the traditional method and one of the auxiliary variables of the new method, the WRF-CMAQ model owns deemed acceptable performance (detailed in Supplementary Table 1) judged by the simulation performance criteria^58^. Compared with the LUR model, the RF model had better performance with higher R^2^ and lower RMSE (Supplementary Fig. 5) and was thus selected for further downscaling and assimilation. The feature importance of the auxiliary variables in the RF model is shown in Supplementary Fig. 6. The PM_2.5_ from WRF-CMAQ and planetary boundary layer height (PBLH) always played vital roles, followed by the meteorological variables like surface temperature (SFCTMP), wind speed at the altitude of 10m (WSPD10), and relative humidity (RH). The high ranking of PBLH shows that the concentration of PM_2.5_ in Beijing had not fallen low enough to decouple the influence of unfavorable meteorological factors, thus owning a high possibility of vertical aggregation to cause heavy pollution. Apart from that, the high contribution of RH and low contribution of WSPD10 in winter indicated that low diffusivity in the horizontal direction and high RH always triggered the secondary formation of PM_2.5_, which is consistent with previous studies^59,60^. The SFCTMP contributed the highest for spring and summer, mainly caused by the high radiant fluxes, possibly leading to the intensified formation of secondary pollution^61^. Contributions of other auxiliary variables such as TOA, land use type, population, and road emission varied in different seasons. The feature importance of TOA is relatively lower since it only has a monthly resolution compared to the hourly-resolution 1 km-PM_2.5_ concentration and meteorological variables. The original raw TOA data has a higher temporal resolution of 10–15 days. The feature importance of TOA is relatively low due to its lower temporal resolution compared to the hourly-resolution 1 km-PM_2.5_ concentration and meteorological variables. Although the original raw TOA data should be able to support higher temporal resolution (10–15 days), it is prone to abnormal values caused by cloud cover or high reflectance underlying surfaces. Therefore, to balance between temporal resolution and data accuracy, we use the median of valid data in each grid within a month as the representative TOA value. However, as the study period extends, the feature importance of TOA is expected to increase due to its close relationship with PM_2.5_ concentration^62–67^.

### Spatial-temporal PM_2.5_ exposure level for the population

**Fig. 3:**
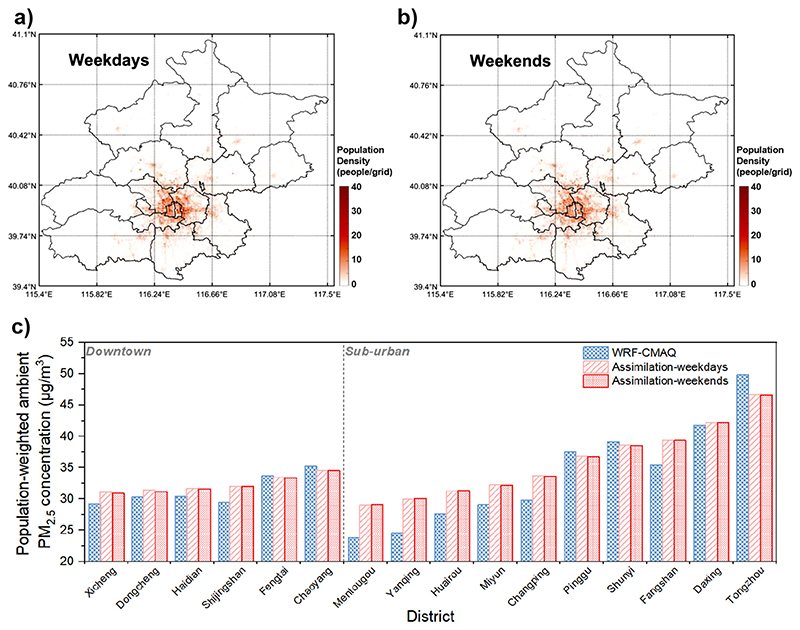
Population distribution and PM_2.5_ exposure pattern in Beijing. **a**, **b** population distribution heat map during weekdays and weekends. **c** Population-weighted ambient PM_2.5_ concentration of 16 Beijing districts during weekdays and weekends.

Population distribution and PM_2.5_ exposure pattern of 16 Beijing districts during weekdays and weekends are shown in Fig. 3. The population density was higher in urban area during weekdays than in weekends, as indicated in sub-figure (a, b) in Fig. 3. The population-weighted ambient PM_2.5_ concentration of the whole Beijing area was 34.6 μg/m^3^ during weekdays and 34.5 μg/m^3^ during weekends, which increased by about 15% compared with the unweighted averaged ambient PM_2.5_ level. Adopting the ultra-high resolution PM_2.5_ concentration field produced a significant difference in the average concentration of population exposure than the WRF-CMAQ model. The main difference of population-weighted ambient PM_2.5_ concentration between the WRF-CMAQ and assimilation result is attributed to the amendment of estimation bias of the WRF-CMAQ result. For the northwestern district with low PM_2.5_ levels, like Shijingshan and Haidian in the urban area and Mentougou, Yanqing, Miyun, Huairou, Changping and Fangshan in sub-urban area, the assimilation lifted the estimated results. In contrast for Tongzhou and Pinggu, the estimated level was lowered. For the other districts in central and southern Beijing, the estimated PM_2.5_ level was moderate and changed little from the WRF-CMAQ to the assimilated result.

**Fig. 4:**
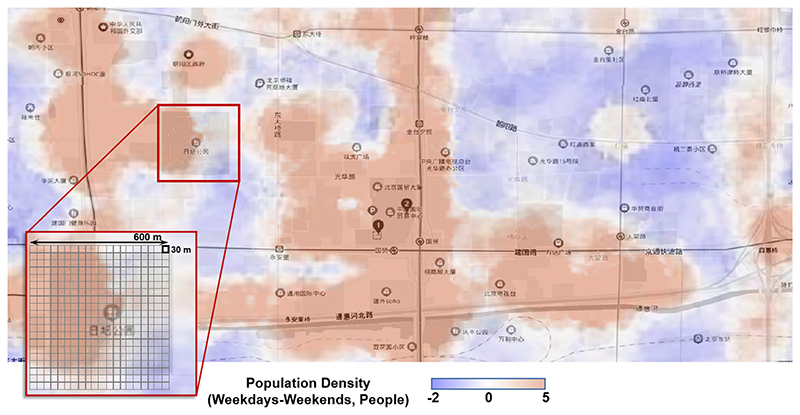
Population distribution difference between weekdays and weekends using 30-m population heat map and building map. Taking the Beijing China World Trade Centre adjacent as an example.

The population-weighted PM_2.5_ concentration shows almost no significant difference between weekdays and weekends, indicating a relatively similar overall population distribution pattern. Nonetheless, the exposure surroundings of the population still exist significant difference during weekdays and weekends. Figure 4 shows an example of the 30-m resolution Population distribution difference between weekdays and week-ends. Taking the Beijing China World Trade Centre adjacent as an example, the population were gathered more in office buildings, streets and subway stations during weekdays, while more in parks, residence areas and railway stations during weekends. Such a difference in exposure pattern could not be reflected using merely the population-weighted ambient PM_2.5_ concentration, and the consideration of exposure micro-environment should be further taken into account.

### Personal PM_2.5_ internal dose assessment

**Fig. 5:**
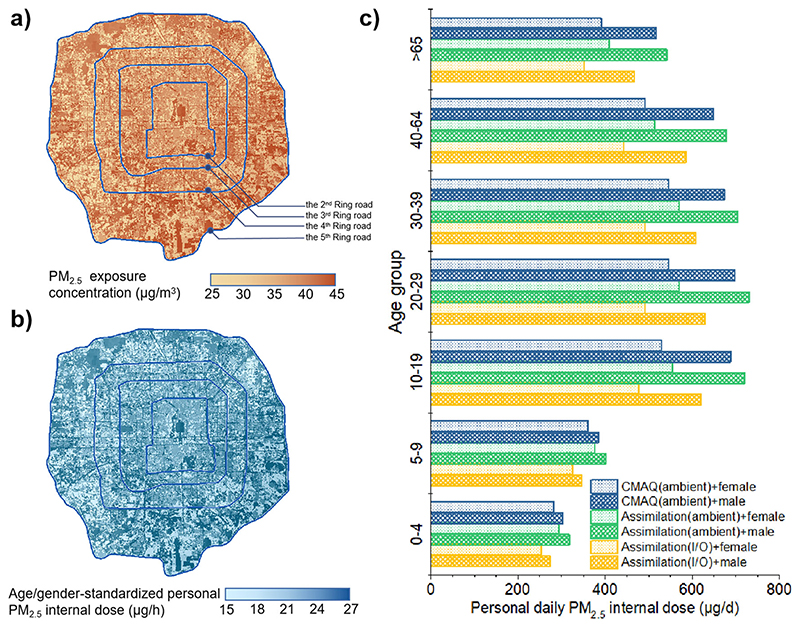
PM_2.5_ exposure pattern of the population within the Fifth Ring Road area in Beijing. **a** PM_2.5_ exposure concentration distribution considering land use type and PM_2.5_ I/O ratio; (**b**) age- and gender-standardized personal hourly PM_2.5_ internal dose distribution; (**c**) comparison of age- and gender-specific average personal daily PM_2.5_ internal dose basing on merely ambient WRF-CMAQ result, ambient assimilation result, and assimilation result considering the I/O exposure difference.

The average indoor PM_2.5_ concentration in Beijing was 26.5 μg/m^3^, ranging from 10.5 μg/m^3^ to 39.5 μg/m^3^. The PM_2.5_ exposure level considering the I/O difference within the Fifth Ring Road is shown in sub-figure (a) in Fig. 5, 26.9 μg/m^3^ indoor and 41.6 μg/m^3^ outdoor on average. As the indoor PM_2.5_ level was estimated by applying the I/O ratio to the ambient level, its trend stayed consistent with the ambient PM_2.5_ map, high in the southeast and low in the northwest. The indoor PM_2.5_ level within the Third Ring Road was moderate and showed no significant difference, but outside the Third Ring Road, the indoor PM_2.5_ level showed an increase in the southeast while a decrease in the north and west. Compared with the result of 57.6 μg/m^3^ from Yang et al.’s study^68^ in 2013–2014 and 38.6 μg/m^3^ from Zuo et al.’s study^69^ in 2017, the indoor PM_2.5_ concentration in this study had dropped a lot as a consequent of air quality improvement. However, it was still over-high compared to indoor PM_2.5_ in other developed countries. For instance, the indoor PM_2.5_ level was mostly lower than 10 μg/m^3^ in England^70^. The indoor environment is one of the main exposure surroundings for most Chinese people, so there is still a great urgency to ease the indoor health burden in our country.

The difference between population distribution and indoor concentration distribution indicated the existence of exposure inequality in the population. On weekdays, people were populated in buildings within the Fourth Ring Road, much higher than the population density in areas outside the Fourth Ring Road. On weekends, the population density in the urban area decreased, and the population spread wider in the sub-urban area. High indoor exposure concentration means that there might be a high exposure risk to the individual health. However, thanks to the low population density in such areas, they would not have a sufficient impact on the population. Conversely, areas with moderate pollution levels owned lower individual exposure risk, but because of the high population density, its impact on the health of the population is more significant. In order to control and reduce exposure risk, the priority of individual and population exposure risk should be further considered during the decision progress. Generally, individual exposure risk evidence was used to formulate the exposure-response relationship and further for formulating the environmental criteria, while the exposure risk for the whole population was taken to formulate the environmental standard. In our study, the exposure risk of the whole population depends mainly on the population distribution. The mobility of the people means that the exposure risk of the whole population was also spatially and temporally dynamic, suggesting the theoretical possibility of dynamic environmental standards can be formulated.

The spatial distribution of age-standardized personal PM_2.5_ internal dose is shown in sub-figure (b) in Fig. 5, developed by the I/O PM_2.5_ exposure level in sub-figure (a). People within the Fifth Ring Road suffered 24.6 μg/h PM_2.5_ from ambient exposure, 22.6 μg/h from residential indoor exposure, and 16.0 μg/h from public indoor exposure on average. The distinguishing of indoor and outdoor exposure allowed us to calculate possibly more accurate personal daily PM_2.5_ internal dose combined with the 30-m population distribution data. The age- and gender-standardized daily PM_2.5_ internal dose was 568.2 μg/d for a single person based on the ambient WRF-CMAQ result, while 594.5 μg/d based on the ambient assimilation result, which increased by about 5%. However, after considering the I/O difference in the exposure level and population distribution, the age-standardized daily PM_2.5_ internal dose was 512.9 μg/d, which decreased significantly by 14%. More specifically, people of different age or gender groups were exposed to different PM_2.5_ doses, as shown in sub-figure (c) in Fig. 5. Males inhaled more PM_2.5_ dose than females because of larger inhalation volume^71^, about 120–150 μg/d above 10-year-old people, while 20 μg/d among children. Estimated with the assimilation result considering the I/O exposure difference, the internal dose of children under 10 was about 250 μg/d. The internal dose of 10 to 64-year-old people was highly similar and the highest during the lifetime, up to 450–600 μg/d, and then began to drop significantly after 65 years old, to about 350–450 μg/d.

Further sensitivity analysis was performed in Supplementary Table 4. As internal doses are divided into indoor and outdoor parts, the sensitivity of total internal dose to the I/O ratio variation demonstrates a sublinear relationship. Within the normal long-term fluctuations, the I/O ratio of residential and public building area changes by 0.2 independently, or fluctuate by ±0.1 simultaneously, the variation in internal dose both does not exceed ±10%. Fluctuations in the population distribution ratio between residential and public buildings do not have a significant impact on the internal dose (within ±2% under ±0.1 ratio changes). All these sensitivity analyses indicated the adoption of the PM_2.5_ I/O ratio was reasonable with low estimating bias.

### Potentials for mortality burden assessment

**Fig. 6:**
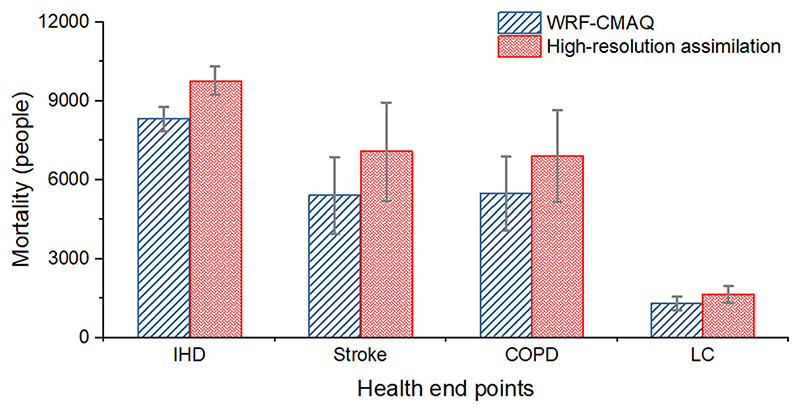
Annual mortality of four certain health endpoints attributed to PM_2.5_ in Beijing, 2019. Annual mortality estimation (with 95% confidential intervals) of ischaemic heart disease, stroke, chronic obstructive pulmonary disease, and lung cancer disease attributable to PM_2.5_ in Beijing based on WRF-CMAQ and high-resolution assimilation results.

The substantial improvement of ambient PM_2.5_ resolution will also largely impact the attributed mortality assessment results. By applying the same GEMM model to the annual WRF-CMAQ result and the ambient high-resolution assimilation result, the estimated annual mortality of IHD, stroke, COPD, and LC disease attributed to PM_2.5_ is shown in Fig. 6. The total mortality (with 95% CI) of the 4 specific health endpoints estimated by WRF-CMAQ result was 20540 (16908-24086) people, while for high-resolution assimilation result was 25462 (20901-29881) people. Stroke was the health endpoint with the highest risk of PM_2.5_ exposure, contributing to almost half of the total attributed mortality, while the lowest was for LC. Overall, the method improved the level of PM_2.5_ by 25.9%, causing the mortality estimation to increase by 24.0%, indicating that the accuracy and resolution improvement of developing ambient PM_2.5_ level may also lead to slightly higher mortality results. It was worth noticing that there is currently no epidemiological evidence to evaluate the pros and cons of using a coarser or higher resolution PM_2.5_ distribution in the mortality burden assessment, not to mention the trade-off between environmental concentration and internal dose. However, in this study, we calculated a series of indicators, including the averaged value, population-weighted averaged concentration, personal internal dose, and mortality estimation within the same frame-work. The evaluation results showed significant differences between the coarser- and higher-resolution result. This shows that the choice of the modelling resolution might have potential impact on health assessment^72^.

In the previous cohort studies, many methods were adopted to derive the PM_2.5_ field because the pollution data were usually not recorded together with the occurrence of health endpoints. The simplest method was directly using the one-hand data, such as the monitoring data from the nearest in-situ stations^73^ or the spatial interpolation method^74^. Some studies also use remote satellite data to derive PM_2.5_ field^75^. However, the spatial resolution of the above method was too coarse, usually 10 km level. Many studies used the CTMs to derive PM_2.5_ field at the 1 km resolution^76,77^, while still too coarse for human activities. To date, some street-level models have been adopted to derive finer PM_2.5_ distribution in order to match with the scale of the activity pattern. For example, the LUR model to derive PM_2.5_ at low concentration levels (<30 μg/m^3^)^78^ might face limitations in high-level regions like Beijing. Moreover, some multi-scale integrated model systems were also adopted^79^, integrating the large and medium-scale CTMs with small scale simulation model under the AirGIS framework. Though with good performance and accuracy, the such kind integrated model relies highly on complex city-level databases and costing enormous computation resources, limiting its application in other cities. Our study provides a 30-m resolution model with good performance (R^2^ = 0.78–0.82) while moderate complexity. It is likely to bring new understanding to the exposure characteristics, making it potential to be adopted in more accurate exposure assessments in the future.

### Uncertainties and perspectives

Still, there are several uncertainties and limitations in this study. The primary source of uncertainty comes from the WRF-CMAQ air quality modeling and RF regression, both of which introduced uncertainties to a different degree, though with acceptable intervals. Furthermore, assumptions were made that the available heat maps accurately represented actual activity patterns of the Beijing population, leading to the underestimation of non-permanent residents in this study. Since various age groups (e.g., elderly individuals) exhibit significantly different activity patterns, they spend varying amounts of time in different areas of buildings. Given that our population activity data cannot be stratified by age, there exists an additional level of uncertainty regarding the exposure of specific population sub-groups. During the estimation of indoor concentration based on the outdoor PM_2.5_, only the I/O ratio without air cleaner was adopted, given the potential complexity of real-life situations and data limitation. Our framework may still underestimate indoor PM_2.5_ concentrations in buildings with complex indoor sources. Studies conducted in environments such as sports arenas and large shopping malls have revealed significant variations in PM_2.5_ I/O ratios, sometimes exceeding 1^80^. Indoor air cleaner plays an essential part in human indoor exposure, there are also available PM_2.5_ I/O ratio with air cleaner working^80^, but currently, there remains no datasets capable for further consideration. Another limitation of the I/O ratio approach is its inability to account for the impact of meteorological factors on I/O PM_2.5_ transport. Researches on office buildings has indicated that factors such as humidity and wind speed can influence the I/O ratio of PM_2.5_. For example, an increase in humidity from less than 40% to 90% can lead to a decrease in the I/O ratio by up to 0.2, and changing of wind speed from less than 1 m/s to over 6 m/s can result in an increase in the I/O ratio of approximately 0.15^81^. Researches also suggested that wind direction can also be a significant influencing factor. However, quantifying the influence of meteorological factors on the I/O ratio is challenging, particularly in densely populated urban areas like Beijing. Street canyon effects, where meteorological conditions within street canyons can significantly differ from those in the urban canopy layer, further complicate the description of short-term I/O ratio variations^82^. Some studies have constructed complex simulation models based on the ventilation characteristics of buildings together with consideration of indoor sources^83^. However, due to data availability constraints, applying these models within the scope of our research remains considerably challenging. Also, this study only provided daily personal indoor PM_2.5_ internal doses in downtown area, for the I/O activity pattern may exist high uncertainty in the sub-urban area with sparse building density.

With further improvements in future air quality, the regional transmission of PM_2.5_ will weaken, while the contribution of local emission sources to PM_2.5_ will increase, leading to an intensification of the spatiotemporal heterogeneity of PM_2.5_ distribution and population exposure. Therefore, it will be necessary to integrate population exposure assessment methods more closely with the effects of local sources. Currently, researches in this area are still limited by technical methods and data availability. Therefore, some prospects have been put forward: (1) The 30-m resolution PM_2.5_ concentration distribution will provide basic data for environmental health assessments; (2) Such method can be applied to multi-year assessment to explore the changing trends in the spatial distribution heterogeneity of PM_2.5_ at the urban scale; (3) This study has proposed a technical framework based on exposure-dose relationship, and future research can further refine in all aspects of this assessment method, e.g. population activity, exposure patterns and I/O PM_2.5_ exposure differences; (4) China has begun to formulate environmental air quality baselines, and understanding the exposure-response relationship based on PM_2.5_ internal dose will provide the most reasonable scientific basis for the baseline formulation.

## Methods

### Study domain and CTM model configuration

**Fig. 7:**
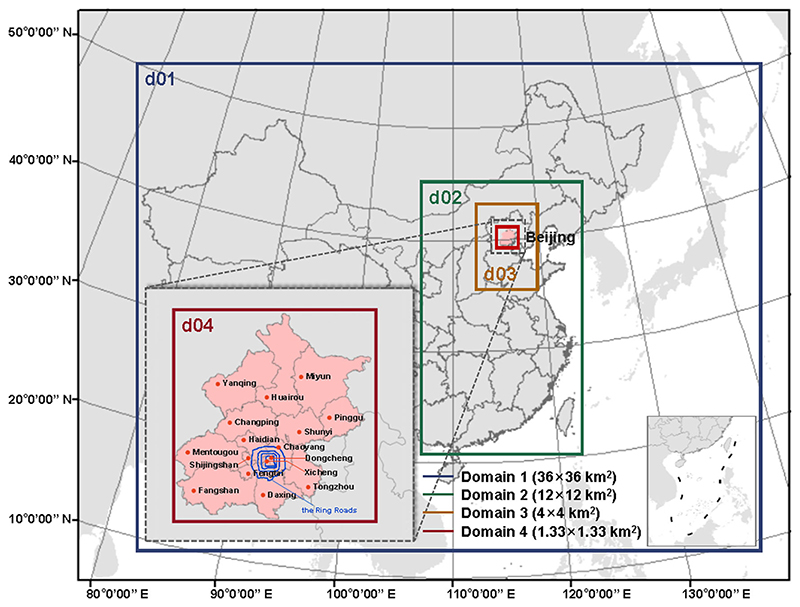
Study domains of 4-level CMAQ modeling and distribution of Beijing districts and the Ring Roads.

The CTMs used in this study were: The Weather Research and Forecasting (WRF) model (version 3.8.1), and the Community Multi-Scale Air Quality (CMAQ) model (version 5.2), which were developed by U.S. National Centre for Atmospheric Research (NCAR) and the U.S. Environmental Protection Agency (EPA), respectively. To simulate PM_2.5_, the WRF–CMAQ system was applied during January, April, July, and October in 2019, with three days of spin-up time for each run, representing corresponding seasons, respectively^84^. To further evaluate the representativeness of the specific months for the corresponding seasons, the similarity of the average value for specific months and corresponding seasons of PM_2.5_ and meteorology conditions in Beijing, 2019 is shown in Supplementary Fig. 1. It exhibits good consistency for the vast majority of the time and monitoring locations, indicating that it is reasonable to use the pollution level of a specific month to represent the whole corresponding season. Beijing, one of the megacities in China, has been taken as the study area. As shown in Fig. 7, the largest modeling domain (d01) covered the area of East Asia and West Pacific, with a temporal resolution of 36 × 36 km^2^. The land-based anthropogenic emissions inventory for mainland China was from the Multi-resolution Emission Inventory for China (MEIC) data at a resolution of 0.25° × 0.25° for the base year of 2015 (MEIC, http://www.meicmodel.org/, last access: 25 October 2018). Base emission of Beijing was gridded as the local emission sources input of the CMAQ model. Here, the local sources refer to the outdoor emission sources within the corresponding modeling domain, with no consideration given to indoor emission sources.

The first guess field and boundary conditions for WRF were generated from the 6 h NCEP FNL Operational Model Global Tropospheric Analyses dataset. The four-dimensional data assimilation (FDDA) was enabled using the NCEP ADP global surface and upper air observational weather data (http://rda.ucar.edu, last access: 25 October 2018). These datasets include all the necessary meteorological parameters required by the WRF model. CMAQ was initialized using the profile file output by the ICON module as the first guess field and boundary conditions, and was pre-run for a period of three days prior to the start date to develop a sufficiently precise monitoring filed. WRF and CMAQ used 32 vertical layers up to 100 hPa, and the lowest layer had a thickness of approximately 37 m. The modeling field and the boundary condition were transferred into a smaller modeling domain with finer resolution (d02, 12 × 12 km^2^) and started another repeated calculation, same as the 3rd (d03, 4 × 4 km^2^) and 4^th^ domain (d04, 1.3 × 1.3 km^2^). Domain 1 covers a larger region in East Asia than the entire country of China. Therefore, the boundary conditions for China were directly derived from the initial meteorological fields provided by WRF. The boundary conditions of CMAQ and the emission inventory follows the same logic as that of WRF in Domain 1. As for the nested grids within the inner layers, the boundary conditions of WRF, the emission and CMAQ for Domain 2 were all derived from the corresponding grid in its parent domain. The same procedure was applied for Domain 3 and 4. In the 4th domain, the Single-Layer Urban Canopy Model (SLUCM)^85^ was coupled with the Noah land-surface model to improve meteorological predictions in the urban area^82^. It assumes the geometric structure of the city is an infinitely long street canyon, and considers the shadowing effect, radiation capture effect and surface reflection effect of the buildings in the street canyon, and specifies the wind profile index, which includes more than 20 parameters such as building height, road width, anthropogenic heat, urban area ratio, and surface albedo.

### Ultra-high-resolution assimilation approach

Since a large proportion of PM_2.5_ in Beijing comes from regional transportation and secondary generation, the level of PM_2.5_ at the county scale is relatively similar^86–88^. The difference of PM_2.5_ in the ultra-fine scale is mainly caused by the spatial-temporal distribution of local emission sources. This difference can be reflected through land use information when there is no high-precision data on sources and sinks. Thus, for assimilation, the auxiliary data set combined 9 major type variables as listed in Supplementary Table 2, containing monitoring data from the national monitoring network, 30-m land use type and satellite data, 1 km-PM_2.5_ level and meteorology derived from CTM results, as well as point of interests (POI), building location, population distribution, traffic emission, and other variables. Ambient air pollution measurements have been conducted routinely by the China Environmental Monitoring Centre (CEME) and Beijing Environmental Monitoring Centre (BJMEMC) since 2013. Hourly PM_2.5_ concentrations were available from 34 of the 35 sites in Beijing (http://113.108.142.147:20035/emcpublish/ and http://zx.bjmemc.com.cn/) from 1 November 2018 to 1 November 2019. The satellite data taken in this study is the top of atmosphere reflectance (TOA) of band 2 of Landsat 8 with 30-m spatial and 16 day-temporal resolution. In order to obtain the complete TOA image covering the whole Beijing area and avoid the influence of extreme values produced by measurement anomalies, the median of the validate TOA value within each 30 × 30 m^2^ grid was chosen and stitched as a whole map, providing a relative spatial trend of monthly concentration distribution at the ultra-high resolution. We realized that the temporal resolution of the satellite TOA data might be low for PM_2.5_ representativeness, but the inclusion of the hourly WRF/CMAQ simulation result, to some extent, had solved the problem, as suggested earlier in the literature^89–91^. The satellite data provide ultra-high-resolution distribution trend of PM_2.5_, and the WRF/CMAQ simulation result provide its temporal changing characteristics. Further information supporting the adoption of satellite data for PM_2.5_ simulation can be found in Supplementary Discussion 1. POI numbers, road line length, and emission intensity were generated from a 1000 m-buffering area around the central 30-m grid to make it more effective in models. All data with a resolution coarser than 30m were further divided into finer meshes with the same properties.

The LUR and RF models were trained and tested to compare the regression performance of the multi-variable dataset. A supervised forward stepwise linear regression was used to develop the LUR model to maximize the adjusted R^2^ value. Due to the total iteration steps were limited by the number of variables, the model was considered to have approximately converged when increasing gradient of R^2^ is not greater than 1%. The RF model was performed using an optimized integrated-tree model, which could be approximately adopted as an RF model. The 10-fold cross-validation and the leave-one-out cross-validation (LOOCV) were taken to evaluate model performance. For 10-fold validation, the training set was divided into 10 random subgroups. One of the subgroups was excluded as a validation set, and the model was recalculated on the remaining nine subgroups. The R^2^, root mean squared error (RMSE) values, and the standard deviation of predicted values from the monitoring data in both the LOOCV and the 10-fold cross-validations were compared to show the validation results. The trained model was applied to a monthly auxiliary dataset, which includes meteorological variables and PM_2.5_ distribution output from WRF-CMAQ averaged at hourly resolution, and other variables with monthly or annual temporal resolution.

Statistical analyses were performed in MATLAB R2021a. ESRI ArcGIS 10.3 was used for geospatial extraction of the auxiliary predictors, and final visualizations of the assimilated PM_2.5_ concentrations by mapping.

### Activity-adjusted population spatial distribution

The population is exposed to different pollution levels according to their activity patterns in various micro-environment during the day-time. In order to capture the location changes of the population more precisely, hourly mobile signaling data from Baidu Smart Eye was adopted. Baidu Smart Eye is a commercial geographic intelligence data platform launched by Baidu Maps with the advancement of technology of cell-phone signal system, describing the population density in real-time. The relative crowdedness in each grid was derived by calculating the population proportion of the grid to the whole Beijing area. An assumption was made that the relative crowdedness of the weekdays and weekends remain similar in other periods of the year, which means, the distribution of the population was assumed unchanged for weekdays and weekends respectively. The population distribution was calculated by the relative crowdedness multiplied by the total population from Worldpop (available from https://www.worldpop.org/geodata/). To date, the resolution of the raw mobile signal data reaches only hundreds of meters^92^, which is even larger than most building scales and is not sufficient to distinguish the I/O exposure differences. Thus, we have to incorporate an inverse distance weighting (IDW) method to interpolate the population distribution to a 30-m resolution in the sparse data area, so that the grid size can be comparable to the building scale. Similarly, due to the lack of supportive data, this study did not further investigate the spatial heterogeneity characteristics of population structure, although there should be differences in gender and age structure among different social places.

### Indoor PM_2.5_ concentration and I/O ratio

The rough resolution of the CMAQ model averages the environmental attributes within 1.33 km. Thus, it is unable to further reflect the differences in indoor and outdoor PM_2.5_ concentration and population distribution at finer scales. Nevertheless, with the newly-derived 30-m resolution PM_2.5_ concentration map, we are able to derive the estimated indoor PM_2.5_ concentration together with the land use type. In the literature, it has been suggested that the indoor PM_2.5_ transported from ambient surroundings can be described by the I/O ratio, infiltration factor, and penetration factor, etc. However, obtaining the infiltration and penetration factors requires a relatively complex model framework, which is difficult to achieve with a comprehensive input dataset covering the entire Beijing area. Additionally, while the I/O ratio is simplified compared to the other mentioned two factors, only a few studies provide representative I/O ratios of the certain kind of buildings^93^ rather than the comprehensive geographic distributions. Thus, limited by data availability, only a unified I/O ratio representing a certain type of buildings was selected from the literature after a review, accounting for the study period and purpose, ambient sampling size, PM_2.5_ level, and comparability with this study, shown in Supplementary Table 3. Typically, the I/O ratio of 0.7 in public building area and 0.9 in residential building area were taken in this study^80^. It is worth noticing that the I/O ratio of PM_2.5_ was influenced by many factors, thus there may exists fluctuations, for example, complexity of indoor sources, air cleaner, etc. Several sensitivity analyses were carried out under different scenarios. The sensitivity of changing in the applied PM_2.5_ I/O ratio to the personal daily PM_2.5_ internal dose was listed in Supplementary Table 4, including sensitivity analysis of: (a) distinguishing PM_2.5_ I/O ratios in residential and public buildings, and (b) population distribution ratio between residential and public buildings, to personal daily PM_2.5_ internal dose.

### Population-weighted ambient PM_2.5_ concentration

Population-weighted PM_2.5_ concentration is widely used to characterize the collective exposure concentration of a population for long-term assessment^94,95^. Based on the 30-m PM_2.5_ concentration and the ultra-high spatial-temporal resolution population distribution, the population-weighted ambient PM_2.5_ concentration of each Beijing district was calculated, shown as the following equation:

(1) $$C_{pop}=\frac{\sum_{i}C_{i}\times P_{i}}{\sum_{i}P_{i}}$$

where:

*i* is a single grid in this district;

*C_pop_* is the annual population-weighted ambient PM_2.5_ concentration in a certain district (μg/m^3^);

*C_i_* is the annual-averaged ambient PM_2.5_ concentration in grid *i* (μg/m^3^);

*P_i_* is the daily averaged population in grid *i*.

### Personal indoor PM_2.5_ internal dose calculation

The quantity of PM_2.5_ inhaled by a person into the lung within a specific time (the internal dose) derived from the PM_2.5_ concentration among the external exposure surroundings (the exposure level) can reflect the PM_2.5_ exposure extent of a person. The higher the personal PM_2.5_ internal dose, the greater the exposure risk a person is likely to suffer. Previous studies have highlighted various factors that significantly impact exposure assessments, such as commuting ways^96^ and age period^97^ that lead to exposure differences. However, due to limited access to fundamental data, we could only use I/O population distribution to characterize the exposure differences, failed to conduct more precise evaluations based on commuting patterns. The 30-m resolution was sufficiently fine in the urban area to classify the I/O exposure difference with high building density. However, in the sub-urban area, 30-m resolution would introduce high uncertainty of the I/O classification due to the small building scale and low building density. Thus, an inhalation model was adopted to develop the personal PM_2.5_ internal dose within the Fifth Ring Road area in Beijing. The age-standardized internal dose of PM_2.5_ was calculated as the following equation:

(2) $$D_{i}=\sum_{j,k}\bar{P_{j,k}}\times {\text{IR}}_{j,k}\times \bar{C_{i}}\times t$$

where:

*D_i_* is the personal internal dose of indoor PM_2.5_ in grid *i* (μg/d);

*i* is a single 30-m grid;

*j* stands for psychological gender, male or female;

*k* stands for age groups divided into 21 groups (5 years as a group, from 0-4, 5-9 to 95-99 and 100+) from the China Statistical Yearbook^98^;

$\bar{P_{j,k}}$ is the average proportion of gender *j* and age group *k*, adopted from the China Statistical Yearbook^98^;

IR*_j,k_* is the inhalation rate of gender j and age group k taken from EPA’s Exposure Factors Handbook^71^ (m^3^/h);

*C_i_* is the estimated PM_2.5_ exposure level in grid *i* (μg/m^3^). For comparison, the ambient CMAQ result, ambient assimilation result, and the assimilation result considering the I/O exposure difference were all adopted individually.

*t* is the daily exposure time (a total 24 h). The I/O ratio of the daily average population within the Fifth Ring Road area reflected the possibility of a person being indoors or outdoors; thus was suitable to be taken as the I/O exposure time ratio of the population. The population within a single 30-m grid was identified as indoor if the land use type was building, otherwise as outdoor (as shown in Fig. 2).

For age- and gender-specific personal daily PM_2.5_ internal dose, the proportion of age and gender was set as 100%, and the inhalation rate was set as the typical value of the age and gender group individually. Detailed information on the population proportion of certain age and gender groups and its mean inhalation rate is listed in Supplementary Table 5.

### Mortality estimation of specific health endpoints

In order to estimate long-term mortality attributable to PM_2.5_ exposure, the epidemiological hazard index (HI) and hazard risk (HR) have been widely used in epidemiological studies. The equation is as follows:

(3) HI=PAF×m×EP

where:

HI the health impact of a specific disease during the assessment period, here annual PM_2.5_-exposure-attributable mortality specifically;

*m* is the age-and-gender standardized cross-sectional mortality (or the so called baseline mortality in the literature) rate of the 25+ population from the GBD study 2019 (available from http://www.healthdata.org/results/data-visualizations);

EP is the exposed population;

PAF (population attributable fraction), calculated by (HR-1)/HR, refers to the potential reduction in morbidity or mortality when the entire population is exposed to the baseline concentration.

The Global Exposure Mortality Model (GEMM) function^95^ was widely taken to calculate HR value for estimating long-term PM_2.5_ exposure-attributed disease burden for the 25+ population. Ischaemic heart disease (IHD), cerebrovascular disease (Stroke), chronic obstructive pulmonary disease (COPD), and lung cancer (LC) are among the most important causes of death covered by HR functions. The International Classification of Diseases 10^th^ Revision (ICD-10) codes of the 4 certain health end points stays consistent with the GBD 2019 Cause-ICD Codes Map (https://ghdx.health data.org/record/ihme-data/gbd-2019-cause-icd-code-mappings). The GEMM developed the exposure-response curve from ambient PM_2.5_ level as follows:

(4) $$\mathrm{HR}(z)=\text{exp}\left\{\frac{\theta \;\text{log}\left(\frac{z}{\alpha }+1\right)}{1+\text{exp}\left\{-\frac{z-\mu }{\nu }\right\}}\right\}$$

where:

*z* is the exceedance ambient PM_2.5_ exposure level over the counterfactual concentration (*C_0_*, the threshold concentration below which no additional health impacts are considered);

HR(*z*) is the hazard ratio of the 25+ population exposed under the exceedance PM_2.5_ exposure level *z* of PM_2.5_, derived from ambient PM_2.5_ level;

*α*, *γ* and *δ* are parameters used to describe the shape of hazard risk curves of different health outcomes^95^.

Details of the parameterization of the GEMM model taken in this study were summarized in Supplementary Table 6. The resolution of the HR, baseline mortality rate and the exposure population was all at the city level, and was all age-and-gender-normalized, here with no specificity across the spatial-temporal dimension. It should be noted that the gridded PM_2.5_ distribution should not be applied to obtain the gridded health risk assessment results. Following the basic developing process and assumptions of the GEMM model, the exposure level scale should be consistent with the baseline mortality rate, here at city-level.

## Supplementary Material

**Supplementary information** The online version contains supplementary material available at https://doi.org/10.1038/s43247-023-01119-3.

Supplementary file

## Figures and Tables

**Fig. 1:**
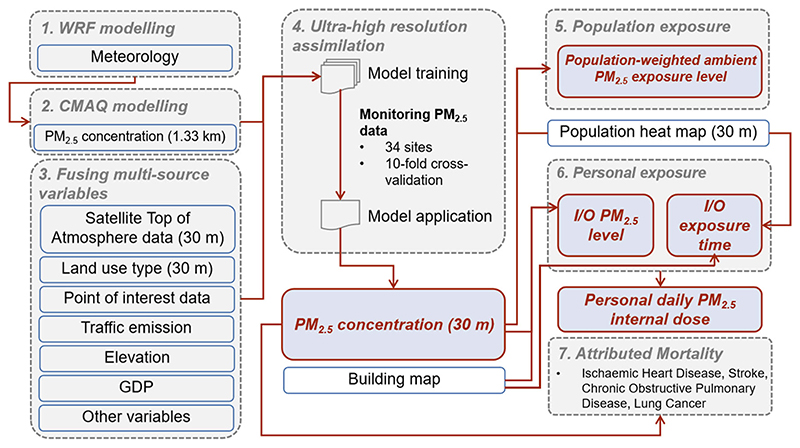
The overall research process of this study. The red part refers to the output dataset from this study, and the gray part refers to the input variables or the processing progress.

## Data Availability

Datasets used for this study can be accessed at: https://zenodo.org/records/10129177. Model outputs are available upon request from the corresponding author Huan Liu (liu_env@tsinghua.edu.cn).

## Code availability

Codes for assimilation are available at: https://zenodo.org/records/10129177.
