# Supplementary material for: Ultra-high-resolution mapping of ambient fine particulate matter to estimate human exposure in Beijing
Source: Commun Earth Environ. Author manuscript; Available in PMC 2023 Dec 21. (PMC7615407; doi:10.1038/s43247-023-01119-3)
Supplement: Supplementary file [file EMS192780-supplement-Supplementary_file.pdf]

Supplementary Information for Ultra-high-resolution mapping of ambient fine particulate matter to estimate human exposure in Beijing

Yongyue Wang[#,1], Qiwei Li[#,1], Zhenyu Luo[1], Junchao Zhao[1], Zhaofeng Lv[1], Qiuju Deng[2], Jing Liu[2], Majid Ezzati[3], Jill Baumgartner[4], Huan Liu[,1], Kebin He[*,1]*

[1]State Environmental Protection Key Laboratory of Sources and Control of Air Pollution Complex, State Key Joint Laboratory of Environmental Simulation and Pollution Control, School of Environment, Tsinghua University, Beijing 100084, China;

[2]Center for Clinical and Epidemiologic Research, Beijing An Zhen Hospital, Capital Medical University, Beijing Institute of Heart, Lung and Blood Vessel Diseases, Beijing 100029, China;

[3]School of Public Health, Imperial College London, London SW72AZ, UK;

[4]School of Population and Global Health, McGill University, Montréal H3A0G4, Canada.

**Correspondence to:* Huan Liu (liu_env@tsinghua.edu.cn) and Kebin He (hekb@tsinghua.edu.cn).
#The authors contribute equally to this paper

Contents

Supplementary Method and Discussions

Supplementary Discussion 1: Relation between satellite measurements and $PM_{2.5}$ simulation

Supplementary Method: Method of two-dimensional discrete Fourier transform and high pass filtering

Supplementary Discussion 2: Result of two-dimensional discrete Fourier transform and high pass filtering

Supplementary Figures

Supplementary Figure 1: Comparison of the average $PM_{2.5}$ pollution levels for specific months and corresponding seasons at 35 monitoring stations in Beijing in 2019

Supplementary Figure 2: Concentration map of seasonal $PM_{2.5}$ in Beijing at 30 m-resolution

Supplementary Figure 3: Energy proportion comparison between CMAQ and assimilation result at different resolution scale on the $PM_{2.5}$ concentration distribution picture using two-dimensional discrete Fourier transform and high pass filtering

Supplementary Figure 4: The performance of high-resolution assimilation method using LUR and RF regression models

Supplementary Figure 5: Feature importance of the auxiliary variables in RF model for four seasons

Supplementary Tables

Supplementary Table 1: Classification and descriptions of the auxiliary variables used in LUR and RF

Supplementary Table 2: Summary of I/O concentration ratios for building area available in studies

Supplementary Table 3: Population ratio and the mean inhalation rate of all age groups in Beijing

Supplementary Table 4: The sensitivity analyses of changing in applied $PM_{2.5}$ I/O ratio to the personal daily internal $PM_{2.5}$ dose

Supplementary Table 5: Exposure-response coefficients and threshold concentrations of $PM_{2.5}$

Supplementary Table 6: The performance of using CMAQ-Model to simulate $PM_{2.5}$ concentration in Beijing

Supplementary References

Supplementary Discussion 1: Relation between satellite measurements and $PM_{2.5}$ simulation

It is widely recognized that satellite data owns high relationship with ground-level $PM_{2.5}$ concentration and incorporating satellite observations into high-resolution $PM_{2.5}$ simulations can significantly improve the accuracy of the simulations. Many studies have successfully retrieved $PM_{2.5}$ concentrations based solely on satellite observations [1-6], with fitting R^2 ranging from 0.6 to 0.9 [6], demonstrating that TOA [3,7] or AOD [8,9] can reflect surface-level $PM_{2.5}$ pollution well. TOA has a positive relationship with AOD, and AOD has a positive relationship with the $PM_{2.5}$ concentration, thus a positive relationship between TOA and $PM_{2.5}$ is expected [10]. Furthermore, satellite observations have been incorporated into machine learning to further improve the accuracy of the simulations. Studies have compared the performance of models with and without satellite observations as auxiliary datasets and found that the inclusion of satellite observations increased R^2 and reduced simulation errors [11]. Bai et al. recently compared the results of using AOD and TOA reflectance to estimate surface $PM_{2.5}$ concentrations, finding that the reflectance-based technique can provide robust $PM_{2.5}$ estimates using random forest machine learning algorithms [1]. In many machine learning models, the satellite measurement data always ranks high in the list of feature importance, indicating that it is very helpful for high-resolution $PM_{2.5}$ predictions [12,13]. Shen's team used deep learning algorithms to create a relationship between $PM_{2.5}$, satellite TOA reflectance, observation angles, and meteorological factors in the Wuhan Urban Agglomeration region, demonstrating a connection between TOA reflectance and $PM_{2.5}$ concentrations [3]. Similarly, Zhang et al. used Landsat 8 satellite remote sensing images, as well as meteorological and time parameters, to specifically estimate PM concentrations using a multilayer perception neural network [14].

Additionally, interference from other pollutants on the remoting of $PM_{2.5}$ was also evaluated. The core principle of satellite remote sensing observation is to estimate atmospheric pollutant concentrations by measuring their absorption of different spectral bands of light. Different pollutants have different spectral absorption characteristics due to their physicochemical properties. A series of studies have shown that the strongest absorption band of ozone is in the ultraviolet spectrum, including the Hartley and Huggins absorption bands, while its absorption in the visible spectrum is relatively weak [15,16]. The characteristic bands of $PM_{2.5}$ are mainly concentrated in the blue, red, and infrared regions [3,17,18]. Currently, a large number of studies and satellite instruments observe ozone using ultraviolet light and observe $PM_{2.5}$ using blue-red infrared light, generating data products such as AOD [19,20]. In comparison, the spectral bands of the two pollutants do not overlap, so high concentrations of ozone in the upper atmosphere will not have a significant impact on the observation of surface-level aerosols.

Supplementary Method: Method of two-dimensional discrete Fourier transform and high pass filtering

The resolution improvement of the image can be measured by calculating the detail energy distribution of the image at each scale of resolution. The image is converted from the spatial domain to the frequency domain using two-dimensional discrete Fourier transform. The original image is essentially a two-dimensional number table or matrix. Converting the spatial domain image to the frequency domain can observe and process the image more intuitively, and is more conducive to frequency domain filtering and other operations. The conversion was operated using the following equation:

$$F(u,v)=\sum_{x=0}^{M-1}\sum_{y=0}^{N-1}f(x,y)e^{-j2\pi(\frac{ux}{M}+\frac{vy}{N})}$$

where:

f(x,y) is the original image, and its size is $M\times N$; x and y are the spatial coordinates of each pixel in the original image (x=0, 1, 2, …, M-1; y = 0, 1, 2, …, N-1);

$F(u,v)$ is the Fourier transform of $f(x, y)$, and its size is also $M\times N$; u and v are the frequency domain coordinate systems after Fourier transform of the original figure (u=0, 1, 2, ..., M-1; v = 0, 1, 2, …, N-1);

j is an imaginary unit.

After the two-dimensional discrete Fourier transform, the original picture will be decomposed into several frequency dividers, each of which will have different energy. The amount of information in each frequency dividers can represent the amount of information in the original picture at each resolution scale:

$$\mathrm{EP}(i)=\frac{|F(u,v)|^2}{\sum_{u=0}^{M-1}\sum_{v=0}^{N-1}|F(u,v)|^2}\times 100\%$$

$$i=\frac{\mathrm{Rsl}}{\sqrt{(\frac{u}{M})^2+\left(\frac{v}{N}\right)^2}}$$

where:

i is the frequency domain scale represented by frequency u and v;

EP (Energy percentage) means the proportion of the information contained in the $F(u,v)$ under frequency domain when the image resolution scale is exactly equal to i.

Supplementary Discussion 2: Result of two-dimensional discrete Fourier transform and high pass filtering

The larger the scale represented by the image frequency division, the lower the frequency division, which tends to describe the overall change trend of the original image; the smaller the scale represented by the image frequency division, the higher the frequency division, and more inclined to describe the detail changes on the small scale of the original image. This operation is similar to waveform filtering. The energy

proportion comparison between CMAQ and assimilation result at different resolution scale on the $PM_{2.5}$ concentration distribution picture using two-dimensional discrete Fourier transform and high pass filtering was shown in Supplementary Figure 3. After the assimilation, similar low frequency bands are basically displayed on the medium and large scales (>40000 m), with rather close band positions and energy ratios, indicating that the assimilation retains the overall spatial distribution trend of CMAQ. On a smaller scale, there is a large difference in energy distribution (<20000 m). The energy distribution of CMAQ is only distributed on the scale of 1.33 km and its integral multiple. Due to its high resolution, few frequency divisions can be decomposed, and the proportion of each division to the total energy is relatively high. However, the energy distribution of the assimilation has reached a finer scale, with much more frequency divisions, but the energy contained in each frequency band is relatively much lower than the CMAQ result, because the information scale under the original rough resolution is further subdivided into a finer resolution.

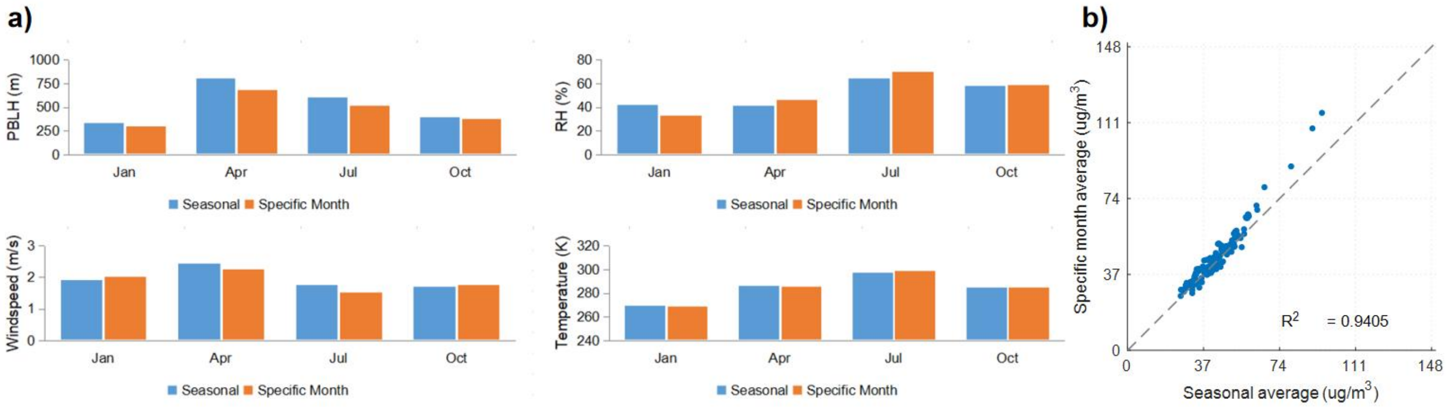

Supplementary Figure 1 Comparison for specific months and corresponding seasons in Beijing, 2019: a) average value of four meteorology variables of the whole area; b) average $PM_{2.5}$ pollution levels at 35 monitoring stations

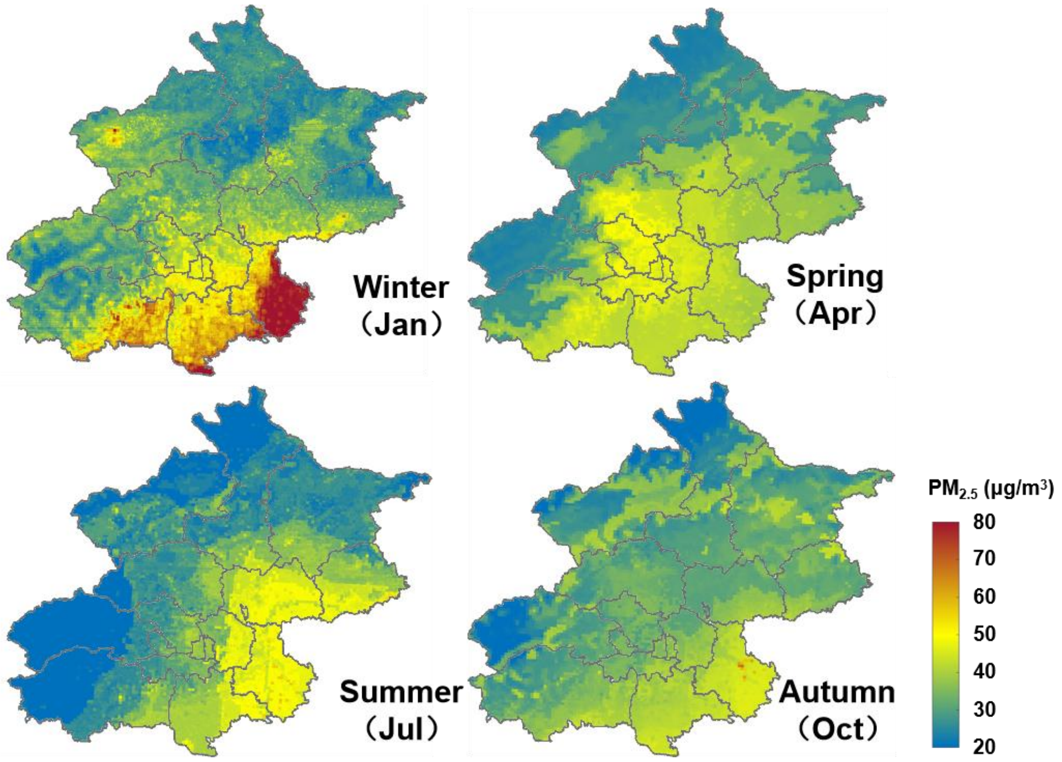

Supplementary Figure 2 Concentration map of seasonal $PM_{2.5}$ in Beijing at 30 m-resolution

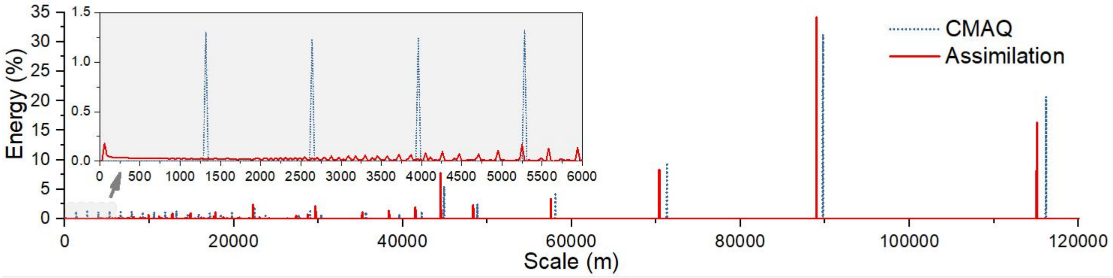

Supplementary Figure 3 Energy proportion comparison between CMAQ and assimilation result at different resolution scale on the $PM_{2.5}$ concentration distribution picture using two-dimensional discrete Fourier transform and high pass filtering

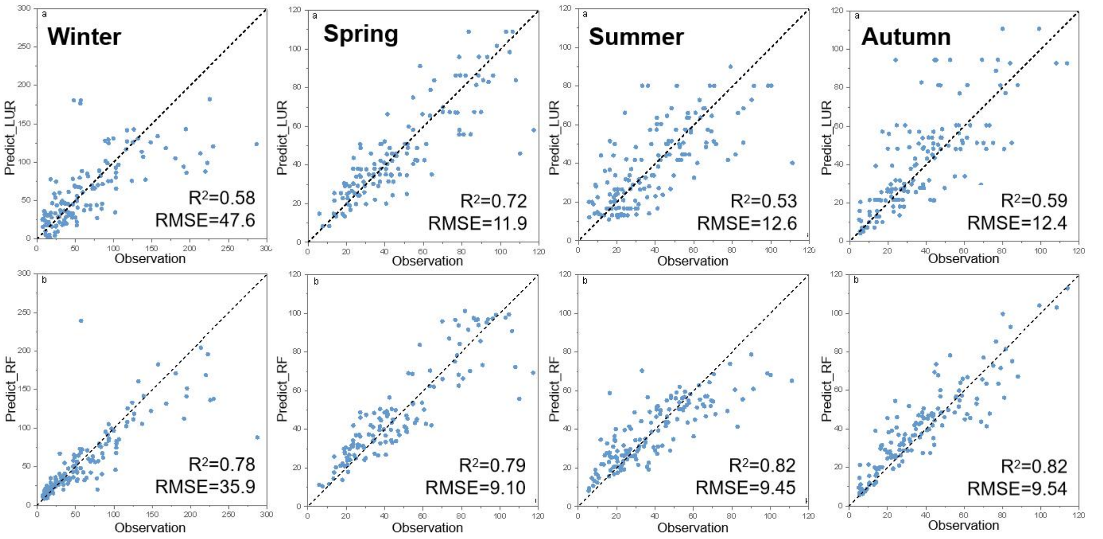

Supplementary Figure 4 The performance of high-resolution assimilation method using LUR and RF models

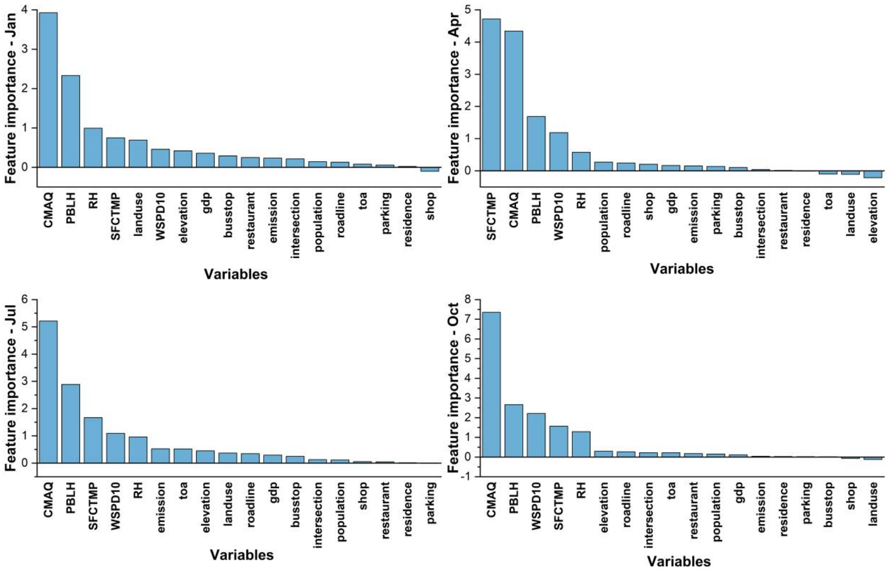

Supplementary Figure 5 Feature importance of the auxiliary variables in RF model for four seasons

Supplementary Table 1 The performance of using CMAQ-Model to simulate $PM_{2.5}$ concentration in Beijing

Statistics	Winter (Jan)	Spring (Apr)	Summer (Jul)	Autumn (Oct)
Data set size	23744	21150	23454	22376
Observation-mean (μg/m3)	58.51	50.46	36.82	38.96
Model-mean (μg/m3)	30.26	30.51	39.47	51.19
MB	-28.10	-19.93	2.95	11.95
NMB	-0.46	-0.40	0.084	0.30
NMGE	0.56	0.49	0.52	0.61
R	0.65	0.69	0.56	0.80
IOA	0.65	0.59	0.52	0.53
MFB*	-0.38	-0.61	0.037	0.060
MFE*	0.62	0.72	0.50	0.56
RMSE	63.14	34.23	26.69	34.52

*Accept simulation performance criteria [21]: |MFB|≤0.6, MFE≤0.75

Supplementary Table 2 Classification and descriptions of the auxiliary variables used in LUR and RF regression models

Category	Predictor Variables	Buffer Radii	Source
**Air Quality Model Output**	● $PM_{2.5}$	-	CMAQ models
**Satellite data**	● Top of Atmosphere reflectance (Seasonal)	-	Landsat 8
**Geographic Character**	● Elevation	-	SRTM
**Land use type**	● Land use mark	-	FROM-GLC
**Traffic Indicator**	● Total Road length (all road, freeway, national road, provincial road and urban road)<br>● Number of intersections<br>● Number of bus stops	1000 m	Gaode POI 2015
**Urbanization Factor**	● Total Residential area<br>● Total Building height<br>● Number of restaurants<br>● Number of shopping center<br>● Number of parking lots	1000 m	Gaode POI 2015
**Emission**	● Road Emission	1000 m	This study
**Meteorology**	● Wind speed<br>● Temperature<br>● RH<br>● PBLH	-	WRF model
**Other potential predictors**	● Population (total)<br>● GDP (average)	-	Landscan 2018

Supplementary Table 3 Summary of I/O $PM_{2.5}$ concentration ratios for building area available in studies

I/O Concentration Ratio	Note	Reference
0.70	0.74 for heating season, and 0.65 for non-heating season; selected for this study.	22
0.61 (0.8-3.1)	Earliest study in Beijing (2013), when outdoor concentration was 171-321 μg/m^3 in average.	23
0.7, 0.9	For public buildings and for residence buildings, respectively. When the average outdoor concentration was 101 (4-364) μg/m^3.	24
0.62	For 41 urban residences in non-heating seasons in Beijing in 2014, when the average outdoor concentration was ~60 μg/m^3.	25
0.4 - 0.9	When the outdoor concentration ranged from 150 to 240 μg/m^3 of Beijing in 2014.	26
1.75, 1.05, 0.76, 0.63	When ambient concentration ranged between 0-33 μg/m^3, 34-65 μg/m^3, 66-129 μg/m^3, and ≥ 130 μg/m^3 for 1092 residential sites of Beijing in the winter of 2013 to 2014.	27
0.56 ± 0.11	When the ambient concentration was 46.8 ± 27.4 μg/m^3, 2366 samples for Chaoyang District.	28
0.26 - 0.92	Require information on the type of filtration of all homes.	29

Supplementary Table 4 The sensitivity analyses of changing in applied $PM_{2.5}$ I/O ratio to the personal daily internal $PM_{2.5}$ dose (Gray grid means reference scenario)

a) Sensitivity analysis of distinguishing $PM_{2.5}$ I/O ratios in residential and public buildings to personal daily internal dose

Air cleaner	Residence I/O	Public I/O	Residence-Public ratio	Internal Dose (μg/d)
N	0.9	0.7	0.6	512.8
N	0.7	0.7	0.6	462.1
N	0.8	0.7	0.6	487.4
N	1.0	0.7	0.6	538.1
N	1.1	0.7	0.6	563.4
N	0.9	0.6	0.6	497.5
N	0.9	0.8	0.6	528.1
N	0.8	0.6	0.6	472.1
N	1.0	0.8	0.6	553.4

b) Sensitivity analysis of population distribution ratio between residential and public buildings to personal daily internal dose

Air cleaner	Residence I/O	Public I/O	Residence-Public ratio	Internal Dose (μg/d)
N	0.9	0.7	0.6	512.8
N	0.9	0.7	0.5	501.5
N	0.9	0.7	0.7	524.0
N	0.8	0.6	0.5	461.3
N	1.0	0.8	0.7	565.0

Supplementary Table 5 Population ratio and the mean inhalation rate of all age groups in Beijing

Age Group [30]	Sex ratio [30]		Mean Inhalation Rate [31] (m^3/day)	
	Male	Female	Male	Female
0-4	1.74%	1.76%	7.60	7.06
5-9	1.27%	1.29%	9.62	9.03
10-14	1.27%	1.28%	17.23	13.28
15-19	2.71%	2.75%	17.23	13.28
20-24	6.67%	6.76%	17.48	13.67
25-29	6.03%	6.10%	17.48	13.67
30-34	4.62%	4.68%	16.88	13.68
35-39	4.36%	4.41%	16.88	13.68
40-44	4.20%	4.25%	16.24	12.31
45-49	4.12%	4.18%	16.24	12.31
50-54	3.41%	3.46%	16.24	12.31
55-59	3.05%	3.09%	16.24	12.31
60-64	1.90%	1.93%	16.24	12.31
65-69	1.31%	1.32%	12.96	9.80
70-74	1.28%	1.29%	12.96	9.80
75-79	0.98%	0.99%	12.96	9.80
80-84	0.51%	0.51%	12.96	9.80
85-89	0.20%	0.20%	12.96	9.80
90-94	0.05%	0.05%	12.96	9.80
95-99	0.01%	0.01%	12.96	9.80
100+	0.001%	0.002%	12.96	9.80

Supplementary Table 6 Exposure-response coefficients and threshold concentrations of $PM_{2.5}$ from GEMM model [32]

Health End Points	θ	Standard error θ	α	μ	ν
IHD	0.2969	0.01787	1.9	12	40.2
Stroke	0.2720	0.07697	6.2	16.7	23.7
COPD	0.2510	0.06762	6.5	2.5	32
LC	0.2942	0.06147	6.2	9.3	29.8

Note: Parameters considering Chinese cohort was taken. C_0 was taken as 2.4 μg/m^3 as GEMM suggested.

Supplementary References:

1 Bai, H., Zheng, Z., Zhang, Y., Huang, H. & Wang, L. Comparison of satellite-based PM2.5 estimation from aerosol optical depth and top-of-atmosphere reflectance. *Aerosol and Air Quality Research* **21**, 200257 (2021).

2 Wang, B. *et al.* Estimate hourly PM2.5 concentrations from Himawari-8 TOA reflectance directly using geo-intelligent long short-term memory network. *Environmental Pollution* **271** (2021).

3 Shen, H., Li, T., Yuan, Q. & Zhang, L. Estimating regional ground-level PM2.5 directly from satellite top-of-atmosphere reflectance using deep belief networks. *Journal of Geophysical Research: Atmospheres* **123**, 13875-13886 (2018).

4 Yang, L., Xu, H. & Yu, S. Estimating PM2.5 concentrations in Yangtze River Delta region of China using random forest model and the Top-of-Atmosphere reflectance. *Journal of Environmental Management* **272**, 111061 (2020).

5 Mao, F. *et al.* Estimating hourly full-coverage PM2.5 over China based on TOA reflectance data from the Fengyun-4A satellite. *Environmental Pollution* **270**, 116119 (2021).

6 Liu, J., Weng, F. & Li, Z. Satellite-based PM2.5 estimation directly from reflectance at the top of the atmosphere using a machine learning algorithm. *Atmospheric Environment* **208**, 113-122 (2019).

7 Yan, X. *et al.* A Spatial-Temporal Interpretable Deep Learning Model for improving interpretability and predictive accuracy of satellite-based PM2.5. *Environmental Pollution* **273**, 116459 (2021).

8 Ma, Z., Hu, X., Huang, L., Bi, J. & Liu, Y. Estimating ground-level PM2.5 in China using satellite remote sensing. *Environmental Science & Technology* **48**, 7436-7444 (2014).

9 Fang, X., Zou, B., Liu, X., Sternberg, T. & Zhai, L. Satellite-based ground PM2.5 estimation using timely structure adaptive modeling. *Remote Sensing of Environment* **186**, 152-163 (2016).

10 Sun, L. *et al.* Aerosol Optical Depth Retrieval over Bright Areas Using Landsat 8 OLI Images. *Remote Sensing* **8** (2016).

11 Xue, T. *et al.* Spatiotemporal continuous estimates of PM2.5 concentrations in China, 2000–2016: A machine learning method with inputs from satellites, chemical transport model, and ground observations. *Environment International* **123**, 345-357 (2019).

12 Xu, Y. *et al.* Evaluation of machine learning techniques with multiple remote sensing datasets in estimating monthly concentrations of ground-level PM2.5. *Environmental Pollution* **242**, 1417-1426 (2018).

13 Kulkarni, P., Sreekanth, V., Upadhya, A. R. & Gautam, H. C. Which model to choose? Performance comparison of statistical and machine learning models in predicting PM2. 5 from high-resolution satellite aerosol optical depth. *Atmospheric Environment* **282**, 119164 (2022).

14 Zhang, B. *et al.* Estimation of pmx concentrations from landsat 8 oli images based on a multilayer perceptron neural network. *Remote Sensing* **11**, 646 (2019).

15 Brion, J. *et al.* Absorption spectra measurements for the ozone molecule in the 350–830 nm region. *Journal of Atmospheric Chemistry* **30**, 291-299 (1998).

16 Chance, K., Burrows, J., Perner, D. & Schneider, W. Satellite measurements of atmospheric ozone profiles, including tropospheric ozone, from ultraviolet/visible measurements in the nadir geometry: a potential method to retrieve tropospheric ozone. *Journal of Quantitative*

Spectroscopy and Radiative Transfer **57**, 467-476 (1997).
17 Zhan, H., Zhao, K., Bao, R. & Xiao, L. Monitoring PM2.5 in the atmosphere by using terahertz time-domain spectroscopy. *Journal of Infrared, Millimeter, and Terahertz Waves* **37**, 929-938 (2016).
18 Steinbrecht, W. *et al.* An update on ozone profile trends for the period 2000 to 2016. *Atmospheric chemistry and physics* **17**, 10675-10690 (2017).
19 Zhang, H. & Kondragunta, S. Daily and hourly surface PM2.5 estimation from satellite AOD. *Earth and Space Science* **8**, e2020EA001599 (2021).
20 Feng, L., Yang, T. & Wang, Z. Performance evaluation of photographic measurement in the machine-learning prediction of ground PM2.5 concentration. *Atmospheric Environment* **262**, 118623 (2021).
21 Boylan, J. W. & Russell, A. G. PM and light extinction model performance metrics, goals, and criteria for three-dimensional air quality models. *Atmospheric Environment* **40**, 4946-4959 (2006).
22 Chi, R. *et al.* Different health effects of indoor- and outdoor-originated PM2.5 on cardiopulmonary function in COPD patients and healthy elderly adults. *Indoor Air* **29**, 192-201 (2019).
23 Song, P. Y., Wanga, L. X., Hui, Y. & Li, R. PM2.5 Concentrations Indoors and Outdoors in Heavy Air Pollution Days in winter. *Procedia Engineer* **121**, 1902-1906 (2015).
24 Deng, G. F. *et al.* Indoor/outdoor relationship of PM2.5 concentration in typical buildings with and without air cleaning in Beijing. *Indoor Built Environ* **26**, 60-68 (2017).
25 Huang, L. H., Pu, Z. N., Li, M. & Sundell, J. Characterizing the Indoor-Outdoor Relationship of Fine Particulate Matter in Non-Heating Season for Urban Residences in Beijing. *Plos One* **10**, ARTN e0138559 (2015).
26 Zhao, L. *et al.* Influence of atmospheric fine particulate matter (PM2.5) pollution on indoor environment during winter in Beijing. *Building and Environment* **87**, 283-291 (2015).
27 Yan-Jun, D. U., Zhang, Y. & Tian-Tian, L. I. Residential indoor exposures of PM2.5 and relationship between indoor and outdoor PM2.5 in winter in Beijing. *Journal of Environment and Health* (2016).
28 Zuo, J. X. *et al.* Using big data from air quality monitors to evaluate indoor PM2.5 exposure in buildings: Case study in Beijing. *Environmental Pollution* **240**, 839-847 (2018).
29 Barkjohn, K. K. *et al.* Using Low-cost Sensors to Quantify the Effects of Air Filtration on Indoor and Personal Exposure Relevant PM2.5 Concentrations in Beijing, China. *Aerosol and Air Quality Research* **20**, 297-313 (2020).
30 Bureau, C. *Statistical yearbook of China (2019)*. (China Statistics Press, 2019).
31 USEPA. *Exposure Factors Handbook (Chapter 6-Inhalation Rates)*. (1996).
32 Burnett, R. *et al.* Global estimates of mortality associated with long-term exposure to outdoor fine particulate matter. *Proceedings of the National Academy of Sciences* **115**, 9592-9597 (2018).
